# Dynamics of pneumococcal nasopharyngeal carriage in healthy children attending a day care center in northern Spain. influence of detection techniques on the results

**DOI:** 10.1186/1471-2334-12-69

**Published:** 2012-03-22

**Authors:** María Ercibengoa, Nerea Arostegi, José M Marimón, Marta Alonso, Emilio Pérez-Trallero

**Affiliations:** 1 Microbiology Department, Hospital Universitario Donostia-Instituto Biodonostia, San Sebastián, Spain; 2 Biomedical Research Center Network for Respiratory Diseases (CIBERES), San Sebastián, Spain; 3 Pediatric Department, Hospital Universitario Donostia-Instituto Biodonostia, San Sebastián, Spain; 4 Department of Preventive Medicine and Public Health, Faculty of Medicine, University of the Basque Country, UPV/EHU San Sebastián, Spain

**Keywords:** *Streptococcus pneumoniae*, Cocolonization, Multiple colonization, Multiplex-PCR, Quellung reaction, Pneumotest-Latex kit

## Abstract

**Background:**

Pneumococcal nasopharyngeal carriage precedes invasive infection and is the source for dissemination of the disease. Differences in sampling methodology, isolation or identification techniques, as well as the period (pre -or post-vaccination) when the study was performed, can influence the reported rates of colonization and the distribution of serotypes carried.

**Objectives:**

To evaluate the prevalence and dynamics of pneumococcal nasopharyngeal colonization in healthy children aged 6-34 months attending a day care center with a high level of hygiene and no overcrowding. The study was performed 3-4 years after the 7-valent pneumococcal vaccine was introduced, using multiple methodologies to detect and characterize the isolates.

**Methods:**

Over 12 months, 25 children were sampled three times, 53 children twice and 27 children once. Three *Streptococcus pneumoniae *typing techniques were used: Quellung, Pneumotest-Latex-kit and multiplex-polymerase chain reaction (PCR). The similarity of isolates of the same serotype was established by pulsed field gel electrophoresis (PFGE) and occasionally the multilocus sequence type (ST) was also determined.

**Results:**

Overall pneumococcal carriage and multiple colonization rates were 89.5% (94/105) and 39%, respectively. Among 218 pneumococci detected, 21 different serotypes and 13 non-typeable isolates were found. The most prevalent serotypes were 19A, 16F and 15B. Serotypes 15B, 19A and 21 were mainly found as single carriage; in contrast serotypes 6B, 11A and 20, as well as infrequent serotypes, were isolated mainly as part of multiple carriage. Most 19A isolates were ST193 but most serotypes showed high genetic heterogeneity. Changes in the pneumococci colonizing each child were frequent and the same serotype detected on two occasions frequently showed a different genotype. By multiplex-PCR, 100% of pneumococci could be detected and 94% could be serotyped versus 80.3% by the Quellung reaction and Pneumotest-Latex in combination (p < 0.001).

**Conclusions:**

Rates of *S. pneumoniae *carriage and multiple colonization were very high. Prevalent serotypes differed from those found in similar studies in the pre-vaccination period. In the same child, clearance of a pneumococcal strain and acquisition of a new one was frequent in a short period of time. The most effective technique for detecting pneumococcal nasopharyngeal carriers was multiplex-PCR.

## Background

*Streptococcus pneumoniae *has a worldwide distribution and causes multiple human diseases, being recognized as one of the most important infectious causes of morbidity and mortality [1,2]. This organism is frequently isolated as a commensal from the upper respiratory tract in the healthy population. Colonization begins very soon after birth and intrapartum colonization has even been described [3]. Most studies have found asymptomatic carriage rates of between 30% and 62% in children under 2 years of age [4]. The wide range of pneumococcal carriage rates is mainly associated with age, socioeconomic living conditions, and differences in sampling, isolation or serotyping techniques [4,5]. The introduction of the 7-valent pneumococcal conjugate vaccine (PCV7) changed the distribution of serotypes causing invasive disease and also produced variation in nasopharynx colonizing serotypes [6]. In Spain, the PCV7 vaccine was introduced in June 2001.

The main objective of this study was to determine the prevalence of pneumococcal nasopharyngeal carriers, its dynamics (variation of isolates), the impact of the PCV7 vaccine on carriage, and the influence of serotyping techniques in carriage studies.

## Methods

### Study design and subjects

This study was performed in healthy children attending a private day care center (DCC) that was chosen for its easy access -very close to our hospital - and the large number of children of medium to high economic class attending. The number of children per 40 square meters never exceeded 20 and each caregiver continuously attended five to seven children. Most children spent 7 hours daily at the center, of which 60-90 minutes were spent playing in groups when physical contact was closer. No child shared eating utensils, pacifiers or similar objects.

All the parents or guardians of the children attending the day care center were invited to take part in the study and written consent was obtained from all who agreed to participate. All participating parents completed a survey that included the children's PCV7 vaccination status and the following risk factors for pneumococcal carriage: diet (breast-feeding, bottled-feeding or a combination of both), number of siblings, parental smoking, previous illnesses requiring hospitalization, and previous antibiotic use. Children were excluded if they had signs of respiratory tract infection or had taken antibiotics within 7 days before sampling. Of the 180 children aged 6 months to 3 years attending the day care center, parental written consent to participate in the study was available in 105 children, who also met the remaining inclusion criteria. The study was approved by the Ethic Committee of the hospital.

Nasopharyngeal samples were obtained by the same pediatrician working at the Pediatric Department of Hospital Donostia. Sampling was performed in three periods: (i) between November 2004 and February 2005 (winter 2004), (ii) between May and June 2005 (spring 2005), and (iii) between November and December 2005 (winter 2005). Immediately after sampling, swabs were inoculated into vials containing skim-milk-tryptone-glucose-glycerol transport and storage medium (STGG) according to the pneumococcal carriage studies protocol of the World Heath Organization [7] and were immediately transferred to the Microbiology Laboratory of Hospital Donostia, where inoculated vials were stored at -80°C until they were cultured.

### Microbiological procedures

After complete thawing and mixing, 100 μl of the inoculated STGG of all samples were cultured onto 5% sheep blood agar and incubated at 37°C for 20-24 hours in a 5% CO_2 _atmosphere. Isolated colonies suspected of being pneumococci were identified using the optochin sensitivity and bile solubility tests. All pneumococcal isolates were serotyped by the Quellung reaction with sera of the Statens Serum Institute (Copenhagen, Denmark), the Pneumotest-Latex kit [8] (Statens Serum Institute), and a multiplex-polymerase chain reaction (PCR) designed "in house" for 62 serotypes. In addition to the isolation of individual pneumococci in a sample, and trying to identify the maximum number of serotypes colonizing a child, a sweep of colonies from the confluent growth on the blood agar plate was collected into 1 mL of STGG and divided into two aliquots: 500 μl were directly used for the serotyping with the Pneumotest-Latex kit and Quellung reaction and the remaining 500 μl were used for nucleic acid extraction using the NucliSENS easyMag automatic extraction platform (bioMérieux, Marcy lÉtoile, France) prior to multiplex-PCR amplification. The multiplex-PCR had an internal control -detection of the autolysin *lytA *gene - to verify species identification [9].

Multiple carriage was considered when two or more *S. pneumoniae *isolates of a different serotype or serogroup were detected in the same sample.

Pulsed-field gel electrophoresis (PFGE) was performed as previously described [10] using *SmaI *restriction endonuclease. Two PFGE patterns were considered different if they had a similarity < 85%. Multilocus sequence typing (MLST) was performed as described on the MLST web site http://spneumoniae.mlst.net/.

Differences in carriage rates and serotype distribution were assessed with Fisher's exact test or the chi-squared test with Yates' correction, as appropriate, using the SPSS software package for windows (version 15.0 software package; SPSS, Chicago, IL, USA). The associations between risk factors affecting colonization and pneumococcal carriage were calculated (Chi-square or Fisher exact test method) considering only the first sample collected from each child.

## Results

A total of 208 samples from 105 children were studied. Three different samples, taken 6 months apart, were collected from 25 children (23.8%). Two samples, collected 6 months apart, were obtained from 53 children (50.5%): 44 children were screened in the first and second sampling periods and nine in the second and third periods. Finally, only one sample was collected in 27 children (25.7%) (Table 1).

**Table 1:** Number of samples investigated and percentage of positive colonization in three distinct time periods

Children sampled on	Total samples	Nov 04/Feb 05	May 05/Jun 05	Nov 05/Dec 05
	**Positive/Investigated (%)**	**Positive/Investigated (%)**	**Positive/Investigated (%)**	**Positive/Investigated (%)**
3 occasions (n = 25)	60/75 (80%)	22/25 (88%)	20/25 (80%)	18/25 (72%)
2 occasions (n = 53)	83/106 (78.3%)	37/44 (84.1%)	39/53 (73.6%)	7/9 (77.8%)
1 occasion (n = 27)	18/27 (66.7%)	2/3 (66.7%)	3/4 (75%)	13/20 (65%)

Total (n = 105)	161/208 (77.4%)	61/72 (84.7%)	62/82 (75.6%)	38/54 (70.4%)

The age distribution of the 105 children when enrolling the study was as follows: 58 (55.2%) were aged 6 to 11 months, 36 (34.3%) were aged 1 to 2 years and 11 (10.5%) were between 2 and 3 years. There was no significant difference in gender distribution: 52.4% were girls (55/105) and 47.6% were boys (p = 0.11).

### Carriage rates and serotype distribution

When only the first sample of each of the 105 children was studied, the carriage rate, considering the results of the three typing techniques, was 81.9% (86/105). Taking into account that 78 children were sampled more than once, 94 (89.5%) of the 105 children were colonized by *S. pneumoniae *at some point during the study. Of these 94 colonized children, 41 (43.6%) carried more than one serotype simultaneously in the same sample; thus, the percentage of multiple carriage among the 105 children studied was 39%. Colonization rates were lower in younger children but there were no statistically significant differences: 86.2%, 94.4% and 99.9% for children aged 6 to 11 months, 1 to 2 years and 2 to 3 years, respectively. No associations were found among rates of colonization in the first sample and any of the risk factors for pneumococcal carriage studied (p > 0.05).

Changes in the pneumococci colonizing each child throughout the study period were common. Among the 76 colonized children with more than one sample, the same serotype was found in both samples in only 13 (17.1%). In another two children a non-typeable isolate was found in two consecutive samples. In three children, the same serotype was found in two samples obtained 12 months apart, although the same serotype was not detected in the intermediate sample in any of these children.

In the remaining children, the same serotype (n = 10) or a non-typeable isolate (n = 2) was found in two samples obtained 6 months apart. The presence of the same serotype in the same child in two different samplings was mainly associated with non-PCV7 serotypes (Table 2, please note the difference between serotypes in the total number of samples and the number of children colonized). No difference in the detection of the same serotype in two samples of the same child was observed for any age group (seven cases of apparent persistence in infants aged 6-11 months, five in children aged 1-2 years and one in children aged 2-3 years) or for a specific serotype. The same serotype was detected in two samples of the same child in five (13.9%) of the 36 of the children colonized with serotype 19A and in two (9.5%) of the 21 children colonized with serotype 16F (p = 1).

**Table 2:** *Streptococcus pneumoniae *serotype distribution in nasopharyngeal samples of carriers collected on three distinct dates

	Time of sampling			Single or multiple carriage per sample				Total samples with this serotype	Serotyping method			Number of children colonized with this serotype
Serotype^a^	Winter 2004	Spring 2005	Winter 2005	Single	With another one	With another two	With another three		Quellung^b^	Pneumotest Latex^c^	Multiplex PCR	
**4**			1	1				1	1	1	1	1
5	1					1		1	0	0	1	1
6A		2			2			2	2	2	2	2
**6B**	13	2		2	9	4		15	15	14	15	14
6C	1						1	1	1	1	1	1
**9V**		2		1	1			2	2	2	2	2
9N		1				1		1	1	1	1	1
10A	3	8	1	8	3	1		12	11	12	12	11
10B	1			1				1	1	1	1	1
11A	12	2		2	8	3	1	14	11	11	14	13
13	1	1				2		2	2	2	2	2
**14**		6	6	8	3	1		12	6	6	12	12
15B	8	10	3	14	5	2		21	20	20	21	20
16F	14	9		8	11	3	1	23	17	17	23	21
19A	9	12	20	30	9	2		41	41	41	41	36
**19F**	2	4		2	3	1		6	6	6	6	5
20	6	9	1	4	9	3		16	6	6	16	16
21	7	6		9	3	1		13	13	13	13	13
22F	2	1			2	1		3	1	1	3	3
23B	6	3	1	7		2	1	10	10	10	10	9
35F	1	1	6	5	2	1		8	8	8	8	7

Total pneumococci serotyped	87	79	39	102	70	29	4	205	175	175	205	191
Non typeable isolates	7	4	2	13				13	13	13	13	11

Total pneumococci detected	94	83	41	115	70	29	4	218			218	

^a ^Serotype: in bold, serotypes included in the seven-valent pneumococcal conjugate vaccine

^b ^Quellung: serotype 6C-specific antiserum, factor 6 d, was not available at the moment of the study and serotype 6C was verified by using factor antiserum 6b

^c ^Pneumotest-Latex kit: the kit used did not allow the identification at the serotype level, being identifications done at the serogroup level

### Sample analysis

One or more pneumococcal types were detected in 161 (77.4%) of the 208 samples collected. A total of 205 different *S. pneumoniae *were serotyped and 13 isolates were non-typeable (Table 2). Multiple colonization was documented in 46 samples: two, three and four different serotypes were detected in 35, 10 and one sample, respectively. The association of serotypes 6B and 21 in the same sample was observed on four occasions and that of serotypes 16F and 20 in five.

Of the 218 pneumococci detected, 30 could only be identified by PCR and all of these were from samples with multiple serotypes.

Overall 21 different serotypes were detected (Table 2). Five serotypes were included in the PCV7 vaccine (4, 6B, 9V, 14, 19F). The most prevalent serotypes were 19A, 16F and 15B. Serotype 19A was found in 41 samples from 36 children: alone on 30 occasions, with another different serotype in nine samples, and together with another two different serotypes on two occasions. Serotypes 19A and 15B were frequently detected as single colonizing serotypes (73.2% and 66.7%, respectively). In contrast, serotype 6B and 11A were detected mainly as part of multiple carriage (86.7% and 85.7%, respectively). Minority serotypes 5, 6A, 6C, 9N, 13, 22F were only detected as part of multiple carriage.

There was no seasonal variation in pneumococcal colonization (Table 1). In cold months, which corresponded to the first and third sampling periods, *S. pneumoniae *was detected in 99 (78.6%) of the 126 samples collected. In warm months, colonization was detected in 62 (75.6%) of the 82 samples studied (p = 0.62).

### Typing techniques

The 208 samples were studied using the three typing techniques (Quellung, Pneumotest and multiplex-PCR) trying to compare the performance of each methodology in the detection and characterization of pneumococcal carriage. When the results of the three techniques were combined, 47 samples (22.6%) were negative, and in the remaining 161 samples, 218 *S. pneumoniae *were detected (Table 2). When the Quellung reaction was used, 175 out of 188 pneumococcal isolates recovered in culture could be serotyped. However, even when the presence of multiple serotypes had been determined by other techniques in a single sample, only 80.3% (175/218) of them could be typed by this technique. When multiplex-PCR was used, 100% of pneumococci could be detected (*lytA *gene positive) and 94% (205/218) were serotyped, these percentages being significantly higher than those identified with the Quellung reaction and the Pneumotest-Latex kit in combination (80.3% 175/218) (p < 0.001).

Thirteen children were colonized with pneumococci of the same serotype and two with two non-typeable pneumococci isolated on different dates. PFGE analysis was performed in 12 pairs of isolates to determine whether isolates with the same serotype detected in the same children but on different dates were the same or different. Apparently similar isolates from eight children showed different PFGE patterns (two couples of non-typeable isolates, three couples of serotype 19A isolates, and one couple each of serotypes 6B, 15B and 23B isolates).

To establish clonality, 35 serotype 19A isolates were studied by PFGE, showing eight different PFGE patterns, although most of these isolates (n = 17) were grouped in one pattern and had the multilocus sequence type (ST) 193. In addition, among eight serotype 11A isolates studied, four different patterns were identified. Six and two different patterns were observed among nine serotype 15B isolates and six serotype 16F isolates studied, respectively. Serotype 14, the most prevalent PCV7 serotype showed three different PFGE patterns.

### Serotype distribution and PCV7

Of the 105 children, 80 (76.2%) had received at least one PCV7 dose (11 with one dose, 27 with two doses, 37 with three doses and five children with four doses). According to the children's vaccination status, no statistically significant difference was observed in the overall pneumococcal carriage rate: 72/80 (90%) in vaccinated children and 23/25 (92%) in non-vaccinated children (p = 1). In contrast, the carriage of vaccine serotypes differed significantly: 18/80 (22.5%) vaccinated children and 11/25 (44%) non-vaccinated children were colonized by a vaccine serotype (p = 0.03).

## Discussion

During childhood, very few children are never colonized by *S. pneumoniae*, while others can be colonized by different serotypes simultaneously [11,12].

Age, socioeconomic conditions and lifestyle influence pneumococcal diseases and carriage [13,14] and are the main non-technical reasons proposed to explain large differences in the rates of acquisition and carriage of pneumococcal isolates [4,5].

Younger children have a more immature immune system than older children, who also acquire immunity via natural exposure. In addition, overcrowding and poor hygiene favour the interchange of infecting microorganisms. In the present study, *S. pneumoniae *colonization peaked around the third birthday but no statistically significant differences were observed among the three subgroups of age. The carriage rate was 89.5%. This high colonization rate obtained from children attending a day care center with a high level of hygiene and no overcrowding was closer to that among underprivileged populations from less developed countries or the suburban populations of developed countries [15-18] than to that of studies from similar European populations [19-21], which reported rates of 14.9%, 51% and 58% for children from Rome, London and Amsterdam, respectively. Such large differences could be explained by genetic background, sampling frequency, seasonality, and other less well-known conditions, [4,5] but sampling and sample processing were probably the most influential factors.

Previous studies have reinforced the importance of methodology in the detection of different serotypes in the same sample [22-25]. Isolates in low densities can be more easily detected using multiplex-PCR and microarray technologies [22-25] than by using the Quellung reaction [26]. A report recently published by Turner et al [25] found that, depending on the laboratory technique employed for *S. pneumoniae *typing, the rates of multiple serotype carriage varied from 11.2% to 48.8%. Our findings demonstrated multiple colonization in 39% of the children (43.6% of colonized children), a high rate compared with most other studies [11,22-25]. Among the three typing techniques used in the present study, the most effective was multiplex-PCR. The Pneumotest-latex kit version used in this study mainly allowed characterization of serogroups rather than of individual serotypes, and consequently this test did not improve the results obtained with the Quellung reaction. Nevertheless, this test was highly useful in detecting co-colonization, shortening the processing times of serotyping and reducing the cost of Quellung reagents. Our findings agree with those of previous studies [22,24] reporting that multiplex-PCR was the most effective pneumococcal serotyping method in nasopharyngeal carriage studies.

Previous studies have reported that carriage duration is usually less than 6 moths [17,25,27]. The design of this study sought to examine three samples per child over 12 months, although temporary drops in attendance at the day care center reduced the number of samples analyzed in some children. Nevertheless, changes in pneumococcal carriage in at least two samples could be studied in 76 out of 94 children. Most of these samples (82.9%) showed a complete change in the type of pneumococci carried and, although the same pneumococci was detected in two samples in 13 children, changes in any of the other serotypes carried by these children were frequent (e.g., in one child serotype 6B was detected together with serotypes 11A and 20 in the first sample but in the second, serotype 6B was detected together with a serotype different to 11A or 20) (Table 3). Moreover, when two isolates of the same serotype were detected in two consecutive samplings, PFGE analysis frequently showed that the serotypes were genetically different. In the three children with the same serotype in two samples obtained 12 months apart, each couple of isolates showed different PFGE patterns.

**Table 3:** Vaccination status and serotypes detected in three consecutive nasopharyngeal samples from 25 healthy children

Children age (months)^a^	PCV7^b ^doses	Winter 2004	Spring 2005	Winter 2005
5	0	19A	19A	negative
6	0	23B	negative	23B
6	0	NT^c^	21	NT
7	0	negative	negative	negative
7	0	6B + 21	NT	negative
10	0	19F + 21	10A	19A
11	0	16F	19A	negative
15	0	23B	15B	19A
16	0	6B + 11A + 20	6B + 16F	14
17	0	negative	23B	14
4	1	19A	35	14 + 19A + 35
5	1	11A + 15B	16F + 19A	35
6	1	negative	21	19A
7	1	21	16F + 19F	19A
10	1	NT	NT	35
10	1	19A	negative	19A
9	2	6B + 19F + 21	10A + 19F	19A
16	2	16F	negative	negative
7	3	21	16F + 19A	14
9	3	6B + 21	19F	19A
10	3	6B + 21	14	negative
12	3	negative	16F + 20	19A
12	3	16F + 35F	15B	NT
15	3	NT	11A + 19A	negative
17	3	16F + 20 + 23B	19A	10A

^a ^Children age (months): when enrolling the study

^b ^PCV7: 7-valent pneumococcal conjugate vaccine

^c ^NT, non typeable but optochin, bile esculin and autolysin *lytA *gene positive

In addition, five of the nine couples of isolates obtained with an interval of 6 months that could be studied also showed different PFGE patterns.

This study has shown a high frequency of changes in the pneumococcal carriage in the same child and reinforces the concept that carriage of a specific isolate is very limited in time.

Twenty-one distinct serotypes were detected, the most prevalent being 19A, 16F and 15B, representing 41.5% of all *S. pneumoniae *serotyped. These serotypes were very different from those found in similar studies in the pre-vaccination period, when the most prevalent serotypes were those included in the PCV7 vaccine [4]. Serotypes 19A and 16F were also the most prevalent serotypes from 2003 to 2005 in Australia [28]. There is limited evidence on the competition or association among the different pneumococcal serotypes in carriage [29,30]. In the present study, serotypes 15B, 19A and 21 were mainly found as single carriage; in contrast, serotypes 6B, 11A and 20, as well as most minority serotypes, were isolated mainly as part of multiple carriage.

Unlike other studies [4,14,31,32], an association between seasonality, family size and passive smoking and colonization, was not found. However, our study has some limitations as the relatively small sample size-number of children and the long periods of time between two samplings.

In the Basque Country, the PCV7 vaccine was not subsidized by the Health Department but 76% of the children attending the day care center were vaccinated. As in other studies [33-36], vaccination status did not affect the global carrier rate but did influence serotype distribution, with non-vaccinated children more often being colonized with vaccine serotypes. The fact that PCV7 serotypes were infrequently found as single colonizing pneumococci, together with the low persistence of these PCV7 serotypes, suggests that PCV7 isolates are carried in low density and indicates a greater effect of the PCV7 vaccine than could be deduced if only the different rate of colonization among vaccinated and non-vaccinated children was considered.

## Conclusions

Despite the introduction of pneumococcal conjugated vaccines, *S. pneumoniae *remains a major cause of morbidity and mortality worldwide, mainly due to the different distribution of serotypes causing disease or replacement of vaccine serotypes. This changing distribution requires effective systems for monitoring the epidemiology of pneumococcal disease and nasopharyngeal colonization studies are very helpful in this task. Different serotypes were found within the same serogroup and different genotypes within the same serotype. This high isolate heterogeneity would have passed unnoticed if the analysis had been less exhaustive, highlighting the importance of molecular methodologies in carriage studies.

## Competing interests

The authors declare that they have no competing interests.

## Authors' contributions

ME conceived the study, participated in its design and in primary data collection, performed microbiology experiments, analyzed the data and helped to draft the manuscript. NA participated in primary data collection and in the epidemiological analysis. MA participated in primary data collection, performed microbiology experiments, and participated in the epidemiological analysis. JMM conceived and participated in the design of the study, performed microbiology experiments, analyzed the global data and helped to draft the manuscript. EPT conceived the study, participated in its design and in project coordination, analyzed the global data and drafted the final version of the manuscript. All authors have read and approved the final version of the manuscript.

## Pre-publication history

The pre-publication history for this paper can be accessed here:

http://www.biomedcentral.com/1471-2334/12/69/prepub
